# The Structure of the Lipid A of Gram-Negative Cold-Adapted Bacteria Isolated from Antarctic Environments

**DOI:** 10.3390/md18120592

**Published:** 2020-11-26

**Authors:** Flaviana Di Lorenzo, Francesca Crisafi, Violetta La Cono, Michail M. Yakimov, Antonio Molinaro, Alba Silipo

**Affiliations:** 1 Department of Chemical Sciences, University of Napoli Federico II, Complesso Universitario Monte S. Angelo, Via Cintia 4, I-80126 Napoli, Italy; molinaro@unina.it; 2 Marine Molecular Microbiology & Biotechnology Institute for Biological Resources and Marine Biotechnologies, CNR-IRBIM Sede di Messina, Spianata San Raineri 86, 98122 Messina, Italy; francesca.crisafi@irbim.cnr.it (F.C.); violetta.lacono@irbim.cnr.it (V.L.C.); mikhail.iakimov@cnr.it (M.M.Y.)

**Keywords:** psychrophiles, Antarctic bacteria, Lipopolysaccharide (LPS), lipid A, structural characterization, MALDI-TOF mass spectrometry

## Abstract

Gram-negative Antarctic bacteria adopt survival strategies to live and proliferate in an extremely cold environment. Unusual chemical modifications of the lipopolysaccharide (LPS) and the main component of their outer membrane are among the tricks adopted to allow the maintenance of an optimum membrane fluidity even at particularly low temperatures. In particular, the LPS’ glycolipid moiety, the lipid A, typically undergoes several structural modifications comprising desaturation of the acyl chains, reduction in their length and increase in their branching. The investigation of the structure of the lipid A from cold-adapted bacteria is, therefore, crucial to understand the mechanisms underlying the cold adaptation phenomenon. Here we describe the structural elucidation of the highly heterogenous lipid A from three psychrophiles isolated from Terra Nova Bay, Antarctica. All the lipid A structures have been determined by merging data that was attained from the compositional analysis with information from a matrix-assisted laser desorption ionization (MALDI) time of flight (TOF) mass spectrometry (MS) and MS^2^ investigation. As lipid A is also involved in a structure-dependent elicitation of innate immune response in mammals, the structural characterization of lipid A from such extremophile bacteria is also of great interest from the perspective of drug synthesis and development inspired by natural sources.

## 1. Introduction

Psychrophiles (or cold-adapted bacteria) are microorganisms able to thrive in permanently cold environments; for others, referred to as psychrotolerants, a larger range of growth temperature is tolerated [1]. In this context, Antarctica, the coldest and most inaccessible continent on the Earth, beside the low temperatures, is characterized by several other extremes including scarcity of nutrients, high or low pH, desiccation and osmotic stress, high levels of UVB radiation and a remarkably variable photoperiod, i.e., from no light to continuous light for 24 h a day [2]. Despite the hostile conditions, prokaryotes are the predominant biomass component in most Antarctic ecosystems including lakes, rivers, ponds, streams, rocks, and soils [3]. This necessarily implies that the ability of psychrophilic prokaryotes to survive and proliferate in Antarctica relies on a number of adaptive strategies aimed at maintaining vital cellular functions even at such prohibitive conditions.

Proteobacteria, a major phylum of Gram-negative bacteria, have been frequently found in Antarctic environments, and represent, with the Actinobacteria, the most abundant phylum isolated from Antarctic soils [4]. Lipopolysaccharides (LPSs), exposed on the external leaflet of the Gram-negative outer membrane, are amphiphilic macromolecules indispensable for viability and survival, as they provide structural stabilization and protection to the whole bacterial envelope, in a dynamic interplay with the external environment [5]. Indeed, under adverse conditions, bacteria can colonize a hostile habitat by modifying their LPS primary structure, in order to reinforce the cell envelope to provide further protection and facilitate adaptation [6]. Consequently, several uncommon structural features have been observed in the LPS of bacteria inhabiting extreme environments, as in the case of cold-adapted bacteria. 

LPSs display a tripartite structural architecture comprising: (i) a highly variable polysaccharide (the O-chain or O-antigen) covalently linked to (ii) an oligosaccharide moiety (the core OS), in turn linked to (iii) the glycolipid part (the lipid A) embedded in the outer leaflet of the outer membrane [5]. With the above three moieties, the LPS is defined as smooth-type LPS (or S-LPS), when lacking the O-chain is designated as rough-type LPS (or R-LPS) [5]. Importantly, LPS is widely known to interact with the mammalian innate immune system, with the lipid A moiety specifically recognized by the host innate immunity receptorial complex TLR4/MD-2 [7]. As a consequence of this interaction and depending on the lipid A fine structure, LPS differently activates the production of host pro-inflammatory cytokines, some causing excessive activation of the TLR4/MD-2 signaling, others exhibiting a weak or no immunopotency. This event can be beneficial to the host, enhancing resistance to infecting microbes, however massive and uncontrolled pro-inflammatory cytokines release can eventually lead to septic shock and multi-organ failure [7]. In this scenario, the search for novel lipid A structures which might possess modulatory activity towards TLR4/MD-2 dependent signaling cascade is considered of high relevance. Herein, LPSs that express uncommon structural features, as the case of psychrophilic bacteria, might act as potential immunomodulators of the TLR4/MD-2 complex. 

From these significant structural and functional perspectives, we here report about the structural characterization of the lipid A from three different psychrophilic bacteria isolated from Terra Nova Bay, Antartica: *Pseudoalteromonas tetraodonis* strain SY174; *Psychromonas arctica* strain SY204b, and *Psychrobacter cryohalolentis* strain SY185. *Pseudoalteromonas tetraodonis* strain SY174 was isolated from platelet ice; *Psychromonas arctica* strain SY204b and *Psychrobacter cryohalolentis* strain SY185 were isolated from marine invertebrates belonged to the genus Holothuria and to the class Hydrozoa, respectively. The three strains were selected because of their abilities to reproduce and rapidly grow at subzero temperatures. Specifically, a generation time of less than 30 days was registered for all these strains at −2 °C, i.e., a temperature comparable to that of Antarctic seawater (field emission scanning electron microscopy micrographs of *P. tetraodonis* SY74 and *P. cryohalolentis* SY185 cultures growing at 0.5 °C are reported in Figure S1). 

All the lipid A structures have been determined by merging information that was attained from the compositional analysis executed on pure LPS with information from a matrix-assisted laser desorption ionization (MALDI) time of flight (TOF) mass spectrometry (MS) investigation executed on the isolated lipid A fractions and directly on bacterial pellets. Finally, an in-depth MS^2^ analysis has been conducted in order to detailly establish the location of the acyl chains with respect to the glucosamine disaccharide backbone of each isolated lipid A fraction.

## 2. Results

### 2.1. Isolation of the LPS and Compositional Analysis of the Lipid A from Cold-Adapted Bacteria

LPS material was extracted from dried bacterial cells and checked via SDS-PAGE after silver nitrate gel staining. This analysis highlighted the smooth-type nature of the LPS from *P. tetraodonis* as proven by the ladder-like pattern in the upper part of the gel, which is diagnostic for the occurrence of high molecular weight species; while a run to the bottom of the gel, typical of a low molecular mass R-type LPS, i.e., an LPS devoid of the O-chain moiety, was observed for *P. cryohalolentis* and *P. arctica*. After their purification, a detailed compositional analysis was performed to establish the fatty acid content (results are summarized in Table 1). This information was key in supporting the following elucidation of the lipid A structures by MALDI-TOF MS and MS^2^ approaches.

In order to investigate the structure of the lipid A portion, an aliquot of each LPS underwent a mild acid hydrolysis, typically performed to selectively cleave the acid labile glycosidic linkage between the core OS and the lipid A moiety. Once the lipid A of each strain was obtained, an aliquot was analyzed to define the nature of the lipid A sugar backbone (i.e., a D-glucosamine disaccharide for all the strains), whereas another aliquot underwent a detailed MALDI-TOF MS and MS^2^ investigation to finally establish the fine structure of the lipid As. Moreover, an aliquot of a lyophilized cell pellet of each psychrophilic strain underwent a direct MALDI-TOF MS analysis (Figures S2–S4). This approach was essential to confirm the structures deduced by analyzing the isolated lipid A fractions, and to exclude any loss of structural data possibly occurring as a consequence of the chemical treatment used to isolated the lipid A (i.e., the mild acid hydrolysis of the LPS).

### 2.2. MALDI-TOF MS and MS^2^ Analysis on the Isolated Lipid A from P. arctica Strain SY204b

**Figure 1:**
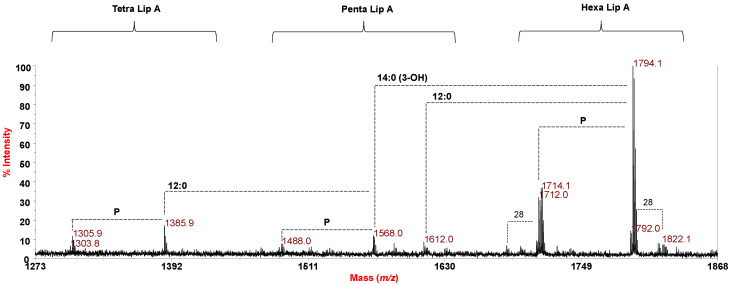
Reflectron MALDI-TOF mass spectrum, recorded in negative polarity, of lipid A from *P. arctica* strain SY204b obtained after acetate buffer treatment. The lipid A species are labelled as Tetra, Penta and Hexa Lip A indicating the degree of acylation. “**P**” indicates the phosphate group.

The negative-ion reflectron MALDI-TOF MS spectrum of the lipid A from *P. arctica* is reported in Figure 1. The main ion peaks and the proposed interpretations of the fatty acids composing the lipid A are reported in Table 2. The spectrum three clusters of signals corresponding to mono- and bis-phosphorylated tetra- to hexa-acylated lipid A species showed in the range of *m*/*z* 1303.8–1822.1, highlighting a heterogenous lipid A blend. Indeed, besides the occurrence of minor peaks differing for 28 amu (i.e., a -CH_2_CH_2_- unit) representing variation in the acyl chains length, peaks differing for 2 amu were also identified, likely indicating the presence of lipid A species also bearing unsaturated acyl moieties, as also shown by compositional analysis (Table 1). Briefly, the main peak at *m*/*z* 1794.1 matched with a bis-phosphorylated lipid A species carrying 14:0 (3-OH) as primary fatty acids, and 12:0 and 14:1 as secondary acyl substituents, the corresponding mono-phosphorylated lipid A species was at *m*/*z* 1714.1. Similarly, mono- and bis-phosphorylated penta-acylated lipid A species lacking one primary 14:0 (3-OH) matched with peaks at *m*/*z* 1488.0 and 1568.0, respectively (Figure 1, Table 2), whereas a penta-acylated form devoid of the 12:0 acyl chain was detected at *m*/*z* 1612.0. The bis-phosphorylated tetra-acylated lipid A species, devoid of one primary 14:0 (3-OH) and the secondary 12:0, was assigned to peak at *m*/*z* 1385.9; the related mono-phosphorylated form was assigned to peak at *m*/*z* 1305.9. Interestingly, the negative-ion MALDI-TOF MS spectrum recorded on the intact *P. arctica* strain SY204b cell pellet (Figure S2) was similar to the above spectrum recorded on the mild acid hydrolysis product (Figure 1); this further confirmed our structural hypothesis and ruled out any loss of structural information.

**Figure 2:**
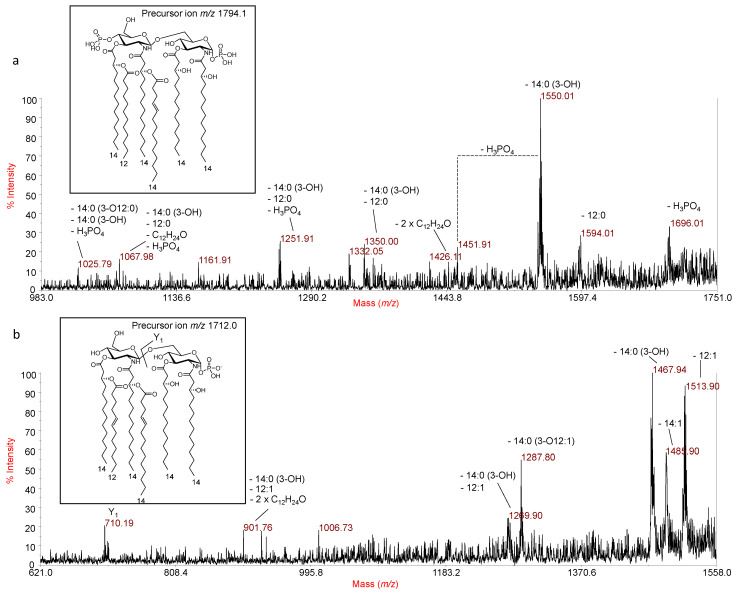
MALDI-TOF MS^2^ analysis of hexa-acylated lipid A species from *P. arctica* strain SY204b. (**a**) Negative-ion MALDI MS^2^ spectrum of precursor ion at *m*/*z* 1794.1, a representative ion peak of the cluster ascribed to hexa-acylated lipid A species decorated by two phosphates. (**b**) Negative-ion MALDI MS^2^ spectrum of precursor ion at *m*/*z* 1712.0, a representative ion peak of the cluster ascribed to hexa-acylated lipid A species decorated by one phosphate. The assignment of main fragments is reported in both spectra. The proposed structure for the lipid A species is reported in each inset. The loss of C_12_H_24_O (184 mass units) is a rearrangement typically occurring on primary 14:0 (3-OH) acyl chains only when their 3-OH group is free, thus contributing to the establishment of the location of the secondary acyl substitution.

A detailed negative-ion MALDI-TOF MS^2^ investigation on various peaks has been performed to define the location of the secondary acyl substituents. The MS^2^ spectrum of the precursor ion at *m*/*z* 1794.1 (Figure 2a), corresponding to a bis-phosphorylated lipid A species carrying four 14:0 (3-OH), one 12:0 and one 14:1, showed an intense peak at *m*/*z* 1550.01 which was attributed to an ion originating from the loss of a 14:0 (3-OH) unit. Less intense peaks were detected at *m*/*z* 1594.01 and *m*/*z* 1696.01 and matched with ions originating from the loss of the 12:0 acyl chain (*m*/*z* 1594.01) and one phosphate unit (*m*/*z* 1696.01), respectively. An ion matching with the sequential loss of one primary 14:0 (3-OH) and the secondary 12:0 unit was also detected at *m*/*z* 1350.00, with the related fragment devoid also of one phosphate unit ascribed to peak at *m*/*z* 1251.91. Importantly, the peak at *m*/*z* 1025.79, matching with a fragment originated from the loss of one phosphate, one 14:0 (3-OH) and a whole unit of a 14:0 (3-OH) fatty acid bearing the secondary acyl substituent 12:0, suggested that the 12:0 was linked to a primary ester-bound acyl moiety. However, to strengthen the structural hypothesis on the exact location and nature of the secondary fatty acids, further MALDI-TOF MS^2^ investigation was performed. The negative-ion MS^2^ spectrum of the precursor ion at *m*/*z* 1712.0, chosen as a reference of a mono-phosphorylated hexa-acylated lipid A species, is reported in Figure 2b. The MS^2^ spectrum, besides furnishing crucial information about the position of the secondary acyl substituents, also provided additional and unreported structural information about *P. arctica* lipid A. In detail, three intense peaks were detected at *m*/*z* 1467.94, 1485.90 and 1513.90 and were assigned as follows: the peak at *m*/*z* 1467.94 was attributed to an ion derived from the loss of a primary 14:0 (3-OH); the ion at *m*/*z* 1485.90 matched with a lipid A fragment originated from the loss of the secondary 14:1 fatty acid, whereas the peak at *m*/*z* 1513.90 was ascribed to an ion derived from the loss of a secondary 12:1 acyl moiety. The occurrence of two unsaturated fatty acids was in agreement with the mass difference of 2 amu with the respect to the species occurring at *m*/*z* 1714.1, i.e., the mono-phosphorylated form of the main lipid A species detected at *m*/*z* 1794.1. Importantly, the occurrence of both the Y_1_ ion (*m*/*z* 710.19) [8], originated from the cleavage of the glycosydic linkage of the disaccharide backbone, and the ion at *m*/*z* 1287.80, which matched with the loss of a whole unit of 14:0 (3-OH) carrying the 12:1 acyl substituent, proved that the secondary fatty acids only decorate the primary acyl chains of the non-reducing glucosamine residue; moreover, these fragmentations suggested that the 14:1 moiety is present as a secondary fatty acid in an acyloxyacyl amide moiety. Therefore, *P. arctica* exhibited a more heterogeneous lipid A than previously reported [6], being composed of species also concomitantly decorated by two unsaturated secondary acyl chains, distributed in a 4 + 2 symmetry with the respect to the glucosamine disaccharide backbone.

### 2.3. MALDI-TOF MS and MS^2^ Analysis on the Isolated Lipid A from P. cryohalolentis Strain SY185

**Figure 3:**
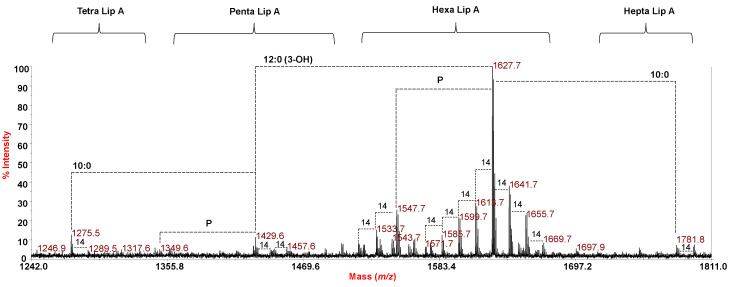
Reflectron MALDI-TOF mass spectrum, recorded in negative polarity, of lipid A from *P. cryohalolentis* strain SY185 obtained after acetate buffer treatment. The lipid A species are labelled as Tetra, Penta, Hexa and Hepta Lip A indicating the degree of acylation. Differences of 14 amu, i.e., a methylene group, are also indicated in the spectrum. “**P**” indicates the phosphate group.

The MALDI-TOF MS spectrum, recorded in negative polarity, of the lipid A isolated from *P. cryohalolentis* LPS is reported in Figure 3, whereas the spectrum recorded on intact bacteria is reported in Figure S3. The high heterogeneity of the lipid A was clearly visible in both spectra, which were similar and showed in the *m*/*z* range 1571.7–1697.9 a main, complex pattern of peaks relative to hexa-acylated lipid A species which differed in the nature of the fatty acids. This was proven by the mass difference of 14 amu occurring between the peaks of the cluster, in agreement with the heterogeneity observed in the lipid compositional analysis (Table 1). This family of peaks, with the main representative at *m*/*z* 1627.7, matched with a bis-phosphorylated lipid A species carrying four primary 12:0 (3-OH), and one 12:0 and one 10:0 as secondary acyl moieties (Table 2). Penta-acylated and tetra-acylated species devoid of one 12:0 (3-OH), or one 12:0 (3-OH) and one 10:0 were identified at *m*/*z* 1429.6 and 1275.5, respectively (Table 2). A less intense cluster of peaks at around *m*/*z* 1781.8 was ascribed to a hepta-acylated lipid A species carrying, in the case of the species at *m*/*z* 1781.8, an additional 10:0 unit (Figure 3 and Figure S3, Table 2).

**Figure 4:**
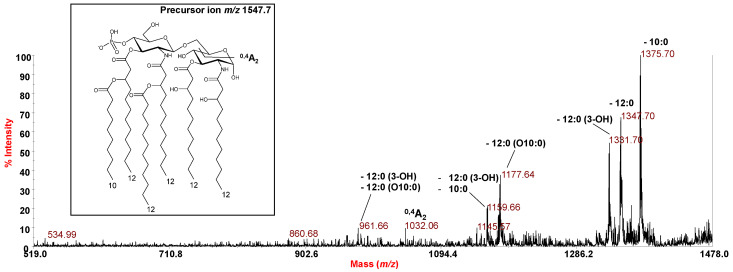
Negative-ion MALDI-TOF MS^2^ spectrum of precursor ion at *m*/*z* 1547.7, main ion peak of the cluster ascribed to hexa-acylated lipid A species decorated by one phosphate. The assignment of the main fragments is reported in the spectrum. The proposed structure for the lipid A species is reported in the inset.

The negative-ion MS^2^ spectrum of precursor ion at *m*/*z* 1547.7 (Figure 4), corresponding to a mono-phosphorylated hexa-acylated lipid A species, confirmed the location of the secondary acyl chains with respect to the glucosamine backbone, that is only on the non-reducing glucosamine unit, as previously reported [9]. This structural hypothesis was corroborated by the observation in the MS^2^ spectrum of an important ion derived from the sugar ring fragmentation ^0,4^A_2_ [8] which clearly indicates the location of the 10:0 and the 12:0 moieties on the non-reducing glucosamine. In parallel, the occurrence of the peak at *m*/*z* 1177.64, ascribable to an ion derived from the loss of a primary ester-bound 12:0 (3-OH) moiety decorated by the secondary 10:0, suggested the location of the latter fatty acid, and likewise of the other secondary acyl chain (12:0) as a substituent of the amide-bound primary 12:0 (3-OH). Similarly, the peak at *m*/*z* 961.66 was attributed to an ion originated from the loss of a 12:0 (3-OH) and a whole unit of 12:0 (3-OH) acylated by the secondary 10:0 moiety. However, the observation of an intense peak at *m*/*z* 1347.70 matching with an ion originating from the loss of the 12:0 unit, suggested that a minor lipid A species might exist with the inverted location of the secondary acyl chains (i.e., 12:0 in an acyloxyacyl ester moiety and 10:0 in an acyloxyacyl amide moiety).

Therefore, as reported [9], *P. cryohalolentis* strain SY185 mainly expresses a highly heterogenous bis-phosphorylated hexa-acylated lipid A species whose primary fatty acids range from 11 to 14 carbon atoms in length, whereas as the secondary acyl chains 10:0, 12:0, 12:1, 14:0, and 15:0 have been found (Table 1).

### 2.4. MALDI-TOF MS and MS^2^ Analysis on the Isolated Lipid A from P. tetraodonis Strain SY174

**Figure 5:**
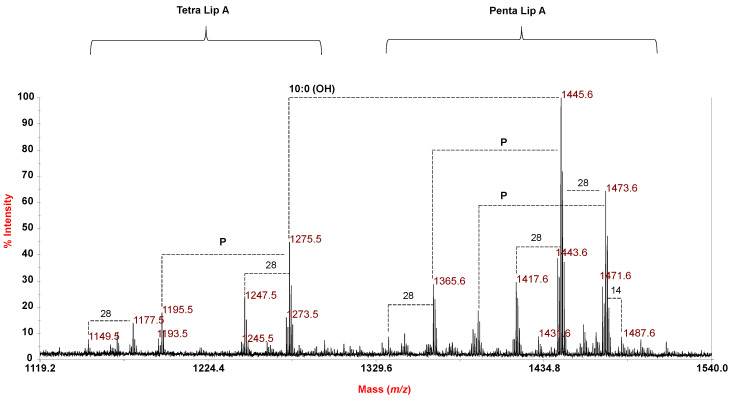
Reflectron MALDI-TOF mass spectrum, recorded in negative polarity, of lipid A from *P. tetraodonis* strain SY174 obtained after acetate buffer treatment. The lipid A species are labelled as Tetra and Penta Lip A indicating the degree of acylation. Differences of 14 and 28 amu are also reported in the spectrum. “**P**” indicates the phosphate group.

The negative-ion MALDI-TOF mass spectrum executed on *P. tetraodonis* lipid A is shown in Figure 5. The well-known heterogeneity of the lipid A was immediately evident, as previously reported by our group for the lipid A structure from other *Pseudoaltermonas* species [10,11], and in full accordance with compositional analysis. The MS spectrum showed two distinct clusters of peaks in the mass range *m*/*z* 1149.5–1487.6, each characterized by the occurrence of mass differences of 14 amu and/or 28 amu. As observed for *P. arctica*, peaks differing by 2 amu were also observed and were attributed to the presence of lipid A, also decorated by unsaturated acyl chains, in agreement with compositional analysis data and with previous reported data [10,11]. Mono- and bis-phosphorylated penta-acylated lipid A species were detected in the mass range *m*/*z* 1337.6 –1501.6, with the main species at *m*/*z* 1445.6 that matched with a bis-phosphorylated penta-acylated lipid A carrying two 12:0 (3-OH) and two 10:0 (3-OH) as primary fatty acids, and one 14:0 as secondary acyl substituent (Figure 5, Table 2). The related mono-phosphorylated species was detected at *m*/*z* 1365.6, whereas a bis-phosphorylated tetra-acylated form devoid of one primary 10:0 (3-OH) was ascribed to the peak at *m*/*z* 1275.5. As observed for the other bacteria analyzed in the present work, the negative-ion MALDI-TOF MS spectrum recorded on the *P. tetraodonis* strain SY174 cell pellet was similar to the one recorded on the isolated lipid A, and is reported in Figure S4.

**Figure 6:**
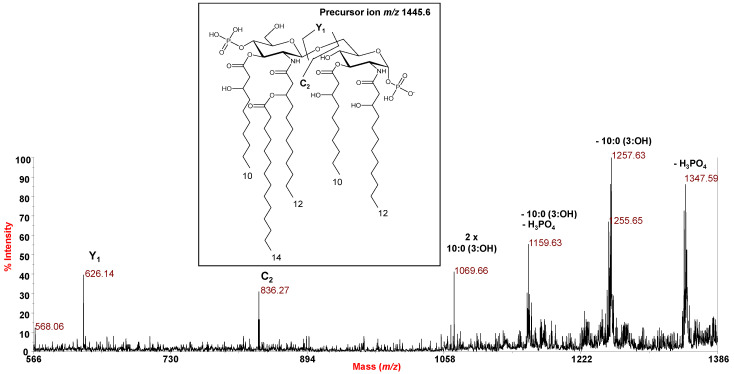
Negative-ion MALDI-TOF MS^2^ spectrum of precursor ion at *m*/*z* 1445.6, main ion peak of the cluster ascribed to penta-acylated lipid A species decorated by two phosphates from *P. tetraodonis* strain SY174. The assignment of main fragments is reported in the spectrum. The proposed structure for the lipid A species is reported in the inset.

**Figure 7:**
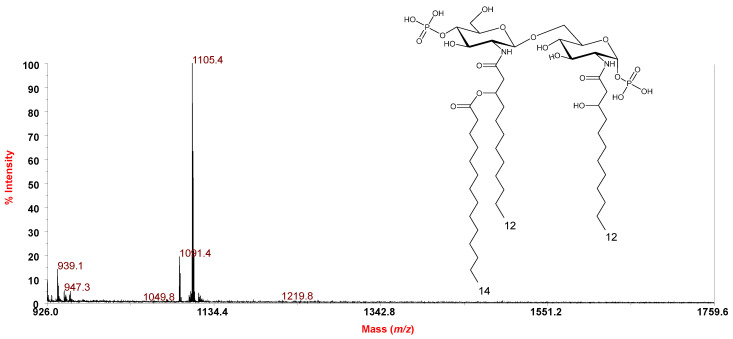
Reflectron MALDI-TOF mass spectrum, recorded in negative polarity, of lipid A from *P. tetraodonis* strain SY174 after treatment with NH_4_OH. The structure of the main ion species is reported in the inset.

The negative-ion MS^2^ investigation of precursor ion at *m*/*z* 1445.6 (Figure 6) showed the occurrence of, among others, important ions originating from the loss of two primary O-linked 10:0 (3-OH) at *m*/*z* 1069.66 which suggested the location of the secondary 14:0 in an acyloxyacyl amide moiety. Moreover, the observation of the two ions at *m*/*z* 626.14 and 836.27 derived from the cleavage of the glycosidic linkage (Y_1_ and C_2_) [8] concurred to locate the secondary acyl substituent on the non-reducing glucosamine unit. However, in order to unequivocally establish the location of the secondary acyl substitution, an aliquot of lipid A, underwent a treatment with NH_4_OH, which selectively removes the acyl and acyloxyacyl esters, leaving the acyl and acyloxyacyl amides unaffected [12]. The negative-ion MALDI-TOF MS spectrum, reported in Figure 7, showed a main peak at *m*/*z* 1105.4 matching with a bis-phosphorylated lipid A carrying the solely primary N-linked 12:0 (3-OH) chains with the secondary 14:0 moiety linked to the primary 12:0 (3-OH) of the non-reducing glucosamine unit (Figure 7), thus definitively confirming the location of the secondary acyl substitution in *P. tetraodonis* lipid A.

## 3. Discussion

The numerous survival strategies adopted by psychrophilic bacteria are considered as an excellent model system to appreciate the mechanisms underlying low-temperature adaptation. Indeed, the capability of some psychrophiles to experience temperatures near to or below the freezing point of water implies the production of cryotolerance-conferring compounds that have attracted the interest of researchers and manufacturers in various industries as potential molecules exploitable in several biotechnological applications [6,13]. In parallel, life in cold habitats also requires an array of adaptive modifications at nearly all levels of the cell envelope architecture and function [14]. Indeed, to endure the cold temperatures, bacterial cell membranes undergo a phase change from liquid to gel, which would dramatically increment the rigidity of the membranes themselves. Therefore, most bacteria modify their membrane lipids to maintain a certain degree of membrane fluidity [14]. Psychrophilic Gram-negative bacteria in this sense greatly modify the structure of the main component of their outer membrane, the LPS molecule, to increment the outer membrane fluidity, thus allowing protein movement, and hence function [6]. Here we reported on the lipid A structures of the LPS from three psychrophilic bacteria isolated from Terra Nova Bay, Antarctica; our analyses provided additional structural information about such an important structural component of the Gram-negative outer membrane component.

As reviewed elsewhere [6], the lipid A from a *P. arctica* strain isolated in the Svalbard islands in the Arctic Ocean was consistent with a blend of species ranging from penta- to hepta-acylated, with a predominant hexa-acylated form composed of the typical bis-phosphorylated diglucosamine skeleton bearing four 14:0 (3-OH), one 14:0 and one cyclopropyl-tetradecanoic acid unit. Here we also found a lipid A structure containing 14:0 (3-OH), as the primary acyl chains, whereas some differences were observed in the nature of the secondary acyl moieties. Indeed, in this study we have highlighted the occurrence of 14:1 acyl residue as well as the concomitant presence of 12:1 as a secondary acyl substitution not previously reported in *P. arctica*. Additionally, neither cyclopropyl-tetradecanoic acids nor hepta-acylated lipid A species [6,9] have been detected in the current study. The discovery of a higher degree of unsaturation found for the lipid A of this *P. arctica* strain isolated from the Antarctica environment is an important structural characteristic which confirms the tendency of cold-adapted bacteria to increment the outer membrane fluidity by modifying and desaturating their membrane lipids, decreasing the lipid packing and thus increasing the membrane fluidity.

As for *P. cryohalolentis*, previous studies on *P. cryohalolentis* K5 (ATCC BAA-1226) strain showed that this bacterium was able to synthesize at least seven major lipid A forms based on a high degree of acyl chains flexibility with a dominant hexa-acylated cluster, and minor clusters of the penta- and hepta-acylated forms [9]. Accordingly, we found a major hexa-acylated lipid A species with the main representative species, detected at *m*/*z* 1627.7 (Figure 3), corresponding to a bis-phosphorylated lipid A carrying four 12:0 (3-OH) and one 12:0 and one 10:0 secondary fatty acids in a 4 + 2 symmetry with the respect to the disaccharide backbone. Minor tetra-, penta- and hepta-acylated species were also here identified. Notably, the *P. cryohalolentis* K5 (ATCC BAA-1226) lipid A structure with a decreasing temperature showed a near-elimination of odd-numbered acyl chains and a shift toward shorter acyl moieties, a structural trend to favor bacterial membrane fluidity [15]. Here the observation in the spectrum (Figure 3) of the occurrence of lesser but significant MALDI-TOF peaks that differ by a single-methylene group flanking the most abundant peak at *m*/*z* 1627.7 demonstrated that this *P. cryohalolentis* strain tends to maintain a certain degree of odd-numbered acyl chains. On the other hand, a shortening of the acyl chains’ moieties with respect to the most abundant lipid A species was observed, as proven by the occurrence of the peaks in the *m*/*z* range 1571.6–1613.7. However, lipid species with longer acyl chains were also detected (*m*/*z* 1641.7–1697.7) in this study. Finally, it is worth underlining that, in accordance with the tendency of cold-adapted bacteria to increase the desaturation of their membrane lipids, here we found that this Antarctic strain of *P. cryohalolentis* produces a lipid A species also carrying an unsaturated secondary fatty acid chain (12:1, Table 1) [9,16].

By contrast, no studies have been reported so far about *P. tetraodonis* lipid A. However, several papers described the structure of the lipid A from other *Pseudoalteromonas* species [10,11] which confirmed and strengthened our results on a heterogenous bis-phosphorylated penta-acylated lipid A species as the main form. Here, the main lipid A species was detected at *m*/*z* 1445.6 that matched with a bis-phosphorylated lipid A species bearing two O-linked 10:0 (3-OH) and two N-linked 12:0 (3-OH) as the primary fatty acids, whereas 14:0 was identified as the secondary acyl substituent. This structural assessment further proved the high heterogeneity of the lipid A species that characterizes *Pseudoalteromonas* sp. LPS because the same peak (at about *m*/*z* 1445.6) was previously observed for other *Pseudoaltermonas* sp. but a different structure was deduced [11]. Importantly, given the controversial data reported in the literature about the exact location of the secondary acyl moiety in the lipid A from *Pseudoalteromonas* species, here we showed that *P. tetraodonis* lipid A is characterized by the occurrence of the secondary fatty acid exclusively on the N-linked primary acyl chain of the non-reducing glucosamine.

It is worth underlining that all of the lipid A structural peculiarities discussed above, especially the occurrence of unsaturated and short acyl chains, are surely of great scientific interest from an evolutionary point of view, improving the comprehension of the molecular mechanisms allowing low temperature adaptation phenomena. Moreover, the discovery of such unusual structural features further proved the never-ending and still undiscovered chemical possibilities arising from microorganisms living in cold environments, thus also becoming incredibly attractive from a chemical point of view. This scenario becomes even more fascinating under the biological/immunological point of view considering that the TLR4/MD-2-mediated immunoactivity of an LPS is strictly related to the lipid A structure. Actually, hypoacylated lipid As (namely lipid A species expressing less than six acyl chains) usually only scantly activate the TLR4-MD-2-mediated immune response [6,7]. Moreover, a 4 + 2 symmetry of the acyl chains with respect to the glucosamine backbone, as in the case of *E. coli* LPS, has been correlated to a more potent immunostimulatory activity, whereas a 3 + 3 symmetry has been related to a reduction in the immunopotency of the whole LPS molecule [6,7]. In this context, previous studies have demonstrated that, despite expressing a bis-phosphorylated hexa-acylated lipid A with a 4 + 2 symmetry, *P. cryohalolentis* LPS exhibits a lower immunostimulatory capacity than *E. coli* LPS [16]; this was attributed to the shorter primary and secondary acyl chains (10–12 carbon atoms) compared with *E. coli* lipid A (12–14), which might not provide an equally efficient LPS activation of the TLR4/MD-2 mediated signaling. Similarly, several LPS from *Pseudoalteromonas* strains have been investigated for their immunological properties, revealing a very weak immunostimulant activity in accordance with the hypo-acylated nature of their lipid A; however, this LPS exhibited an interesting inhibitory capacity towards the toxic effects of the *E. coli* LPS [17]. The observation that, depending on the structure of the lipid A, LPSs isolated from psychrophilic bacteria are able to modulate the host immune response, in addition to the unlimited sources from which it is possible to isolate such microorganisms, represents an incentive for an in-depth investigation of cold-adapted Gram-negatives as a potential never-ending basin of natural immunomodulatory molecules. Therefore, a detailed study of the immunological properties of the particular LPS/lipid A here defined would be essential to improve the current knowledge about the role of the exact location and nature of the fatty acids in the LPS structure-immunoactivity relationship.

## 4. Materials and Methods

### 4.1. Bacterial Strains Isolation and Growth

Sampling was done during the XXXIII Antarctic Expedition in October/December 2017 in Terra Nova Bay, Antarctica. A sample of platelet ice was obtained from the bottom of 1.5 m thick and 2 m high ice core collected from Tethys Bay (74°40′01.3″ S and 164°11′58.6″ E); invertebrates were collected by Scuba-diving (74°41′24.5″ S and 164°06′15.4″ E). For cultivation, 1 mL of melted platelet ice was inoculated in 9 mL of sea water based medium (SWBM) (g/L^−1^: 0.27 NH_4_Cl, 0.089 Na_2_HPO_4_, 1.30 TAPSO, 0.002 FeCl_2_ × 4H_2_O, 0.002 Fe NH_4_ citrate) supplemented with 300 mg (yeast extract, peptone, casamino acids, sucrose). Cultures were incubated at a 4 °C temperature for one week. 100 μL of positive enrichment was spread onto agar plates of the same medium. Invertebrates were rinsed in sterile sea water and one cm^3^ of each sample was homogenized in 10 mL of SWBM medium in a sterile mortar. Then a ten-fold serial dilution was carried out to obtain dilutions varying from 10^−1^ to 10^−6^. 100 μL of each dilution was spread onto plates of SWBM medium supplemented with 1.5 g (Yeast extract, peptone, casamino acids, sucrose). Cultivation was performed at 4 °C and pure cultures were obtained on plates. The colony transfer was repeated at least twice before the cultures were considered pure. Isolates were checked microscopically and by 16S rRNA sequencing. To obtain a sufficient amount of biomass for LPS isolation and characterization, growth of each strain was performed at 4 °C in ten 6 L flasks. The cultures were incubated for 5 days with periodic shaking.

### 4.2. LPS Isolation and Purification

The LPS from each bacterial strain was extracted by a modified enzyme-phenol-water protocol [18]. Each LPS was obtained as a precipitate after an ultracentrifugation step (200,000× *g*, 4 °C, 16 h). Then, an enzymatic digestion was executed to remove possible cell contaminants by using DNase (DN25-Sigma Aldrich^®^, St. Louis, MO, USA), RNase (R5503-Sigma Aldrich^®^), and protease (P4630-Sigma Aldrich^®^). The digested materials were then again extensively dialyzed (Spectra/Por^®^, Fisher Sci. Leicestershire, UK, cut-off 12–14 kDa) against distilled water. In order to remove any phospholipids that were possibly present, all the LPS underwent several washes with a mixture of CHCl_3_/CH_3_OH (1:2, *v*/*v*) and CHCl_3_/CH_3_OH/H_2_O (3:2:0.25, *v*/*v*). After the removal of organic solvents and repeated cycles of lyophilization, an SDS-PAGE followed by gel staining with silver nitrate [19] was performed to establish the nature and the degree of purity of the extracted materials.

### 4.3. Chemical Analyses

The total fatty acid content was established by treating each LPS with 4 M HCl (100 °C, 4 h), followed by a treatment with 5 M NaOH (100 °C, 30 min). After the adjustment of the pH, an extraction in chloroform led to the collection of the fatty acids, which were then methylated with diazomethane and analyzed by a Gas Chromatography Mass Spectrometry (GC-MS). The ester-bound fatty acids, analyzed by GC-MS, were obtained after treatment with aqueous 0.5 M NaOH in CH_3_OH (1:1, *v*/*v*, 85 °C, 2 h), followed by acidification of the products, extraction in chloroform and methylation with diazomethane. Furthermore, an aliquot of each LPS fraction was also methanolized with 1.25 M HCl/CH_3_OH (80 °C, 16 h). The mixture was extracted three times with hexane. The hexane layer, containing the fatty acids as methyl esters derivatives, was then analyzed by GC-MS. The absolute configuration of the fatty acids was established as previously reported. Briefly, the 3-hydroxy fatty acids were released after treatment with 4 M NaOH (100 °C, 5 h), converted into the 3-methoxy acid L-phenylethylamides, and then analyzed by GC-MS [20]. The comparison of the retention times of authentic L-phenylethylamides of various standard fatty acids with those derived from the examined LPSs allowed the assignment of the (R) configuration to all of the fatty acids composing the lipid A from the three psychrophilic bacteria. The analyses were all executed on an Agilent Technologies gas chromatograph 6850A equipped with a mass selective detector 5973N and a Zebron ZB-5 capillary column (Phenomenex, Torrance, USA, 30 m × 0.25 mm internal diameter, flow rate 1 mL min^−1^, He as carrier gas). The following temperature program was employed for the lipid analysis: 140 °C for 3 min, 140 °C → 280 °C at 10 °C min^−1^.

### 4.4. Isolation of the Lipid A Fractions

An aliquot of each purified LPS was treated with acetate buffer (pH 4.4, 2 h, 100 °C) in order to separate the lipid A portion from the saccharide part of the LPS. A mixture of chloroform and methanol was added to the hydrolysis product to obtain a CHCl_3_/CH_3_OH/hydrolysate 2:2:1.8 (*v*/*v*/*v*) ratio. The mixture was then shaken and centrifuged. The chloroform phase, containing the lipid A, was collected and washed with the water phase of a freshly prepared Bligh/Dyer mixture (CHCl_3_/CH_3_OH/H_2_O, 2:2:1.8) [21]. The organic phases were pooled, dried, and analyzed by MALDI-TOF MS (SCIEX, Concord, ON, Canada). In order to establish the nature of the sugar backbone of each lipid A, an aliquot of each lipid A fraction also underwent a methanolysis (1.25 M HCl/CH_3_OH, 80 °C, 16 h) followed by acetylation (80 °C, 20 min) and GC-MS analysis [22].

### 4.5. MALDI-TOF Mass Spectrometry

All the MS and the MS^2^ experiments were performed both in linear and reflectron mode, negative ion polarity on an ABSCIEX TOF/TOF 5800 Applied Biosystems (Foster City, CA, USA) mass spectrometer equipped with an Nd:YAG laser (λ = 349 nm), with a 3 ns pulse width and a repetition rate of up to 1000 Hz, and also equipped with delayed extraction technology. Lipid A fractions were dissolved in CHCl_3_/CH_3_OH (50:50, *v*/*v*). The matrix solution was 2,4,6-trihydroxyacetophenone in CH_3_OH/0.1 % trifluoroacetic acid/CH_3_CN (7:2:1, *v*/*v*/*v*) at a concentration of 75 mg/mL [23,24]. Bacterial pellet for MALDI preparation was treated as previously described [25,26] and the matrix solution was prepared by dissolving 2,5-dihydroxybenzoic acid (DHB) at a final concentration of 10 mg ml^−1^ in CHCl_3_/CH_3_OH (9:1, *v*/*v*) [25,26]. 0.5 μL of the sample and 0.5 μL of the matrix solution were deposited onto a stainless steel plate and left to dry at room temperature. Each spectrum in the MS experiments was a result of the accumulation of 1500 laser shots, whereas 5000–7000 shots were summed for the MS^2^ spectra. Each experiment was performed in triplicate.

## 5. Conclusions

In the present paper we reported on the structural characterization of the LPS lipid A from three taxonomically different psycrophilic Gram-negative bacteria isolated from Terra Nova Bay, Antarctica. All the three cold-adapted bacteria, *P. tetraodonis* strain SY174, *P. arctica* strain SY204b, and *P. cryohalolentis* strain SY185 displayed structural features in their lipid As, likely supporting and favoring their ability to live and proliferate in Antarctica, the coldest continent on Earth. Among others, a high level of heterogeneity, both in the acylation and in the phosphorylation degree, has been observed for all the analyzed lipid As. In addition, in accordance with the tendency of psychrophiles to increase the desaturation of their membrane lipids, unsaturated fatty acids were detected as structural constituents of all the three lipid As. In particular, *P. arctica* strain SY204b showed an even more heterogenous lipid A than previously reported [6], being characterized by the occurrence of species also concomitantly carrying two unsaturated acyl moieties. Likewise, a tendency to decrease the length of the acyl chains, in order to maintain membrane fluidity at cold temperatures, was also observed, as proven by the occurrence of fatty acids ranging from 10 (*P. cryohalolentis* strain SY185 and *P. tetraodonis* strain SY174) to maximum 13 (*P. tetraodonis* strain SY174), 14 (*P. arctica* strain SY204b), or 15 (*P. cryohalolentis* strain SY185) carbon atoms in length. Finally, the structure of the lipid A from *P. tetraodonis* strain SY174 has been here described for the first time, and confirmed the high heterogeneity of the tetra- and penta-acylated lipid A species found in other *Pseudoalteromonas* species [10,11]. This study further supports and strengthens data describing the existence of lipid A modification systems that are often regulated by environmental conditions [27], and underlines the capability of extremophile bacteria to modify their outer membrane lipid components to deal with the detrimental stressors of the surrounding environment.

## Figures and Tables

**Table 1:** Fatty acid content of the lipopolysaccharide (LPS) isolated from the three cold-adapted bacteria examined in the current study. “+” and “-” indicate the presence and absence of the fatty acid in the lipid A, respectively. All the strains are characterized by a disaccharide of D-glucosamine as the lipid A sugar backbone. For the unsaturated acyl chains the position of the double bond or the stereochemistry remain to be defined.

Fatty Acid Component	*P. arctica* Strain SY204b	*P. cryohalolentis* Strain SY185	*P. tetraodonis* Strain *SY174*
*3-hydroxylated fatty acids*			
10:0 (3-OH)	-	-	+
11:0 (3-OH)	-	+	+
12:0 (3-OH)	-	+	+
13:0 (3-OH)	-	+	+
14:0 (3-OH)	+	+	-
*non-hydroxylated fatty acids*			
10:0	-	+	+
11:0	-	-	+
12:0	+	+	+
13:0	-	+	+
14:0	+	+	-
15:0	-	+	-
*non-hydroxylated unsaturated fatty acids*			
12:1	+	+	+
13:1	-	-	+
14:1	+	-	-

**Table 2:** The main ion peaks observed in the MALDI-TOF MS spectra reported in Figures 1, 3 and 5, the predicted mass and the proposed interpretation of the substituting fatty acids and phosphates on the lipid A backbone. The observed masses reported in the table are compared to the calculated molecular weight (predicted mass, Da) of each ion based on the proposed lipid A structures.

***P. arctica* Strain SY204b**			
**Predicted Mass (Da)**	**Observed Ion Peaks (*m*/*z*)**	**Acyl Substitution**	**Proposed Fatty Acid/Phosphate Composition**
1794.20	1794.10	Hexa-acyl	HexN^2^P^2^[14:0(3-OH)]^4^ (12:0) (14:1)
1792.18	1792.09	Hexa-acyl	HexN^2^P^2^[14:0(3-OH)]^4^ (12:1) (14:1)
1714.23	1714.11	Hexa-acyl	HexN^2^P[14:0(3-OH)]^4^ (12:0) (14:1)
1712.21	1712.08	Hexa-acyl	HexN^2^P[14:0(3-OH)]^4^ (12:1) (14:1)
1612.03	1612.05	Penta-acyl	HexN^2^P^2^[14:0(3-OH)]^4^ (14:1)
1568.00	1568.04	Penta-acyl	HexN^2^P^2^[14:0(3-OH)]^3^ (12:0) (14:1)
1488.04	1488.04	Penta-acyl	HexN^2^P [14:0(3-OH)]^3^ (12:0) (14:1)
1385.84	1385.91	Tetra-acyl	HexN^2^P^2^ [14:0(3-OH)]^3^ (14:1)
1305.87	1305.91	Tetra-acyl	HexN^2^P [14:0(3-OH)]^3^ (14:1)
***P. cryohalolentis* Strain SY185**			
**Predicted Mass (Da)**	**Observed Ion Peaks (*m*/*z*)**	**Acyl Substitution**	**Proposed Fatty Acid/Phosphate Composition**
1782.16	1781.78	Hepta-acyl	HexN^2^P^2^[12:0 (3-OH)]^4^ (12:0) (10:0)^2^
1628.02	1627.68	Hexa-acyl	HexN^2^P^2^[12:0 (3-OH)]^4^ (12:0) (10:0)
1642.04	1641.70	Hexa-acyl	HexN^2^P^2^[12:0 (3-OH)]^3^ [13:0 (3-OH)] (12:0) (10:0)
1548.06	1547.74	Hexa-acyl	HexN^2^P [12:0 (3-OH)]^4^ (12:0) (10:0)
1429.86	1429.57	Penta-acyl	HexN^2^P^2^[12:0 (3-OH)]^3^ (12:0) (10:0)
1275.73	1275.46	Tetra-acyl	HexN^2^P^2^[12:0 (3-OH)]^3^ (12:0)
***P. tetraodonis* Strain SY174**			
**Predicted Mass (Da)**	**Observed Ion Peaks (*m*/*z*)**	**Acyl Substitution**	**Proposed Fatty Acid/Phosphate Composition**
1445.86	1445.59	Penta-acyl	HexN^2^P^2^[12:0 (3-OH)]^2^ [10:0 (3-OH)]^2^ (14:0)
1473.89	1473.61	Penta-acyl	HexN^2^P^2^[12:0 (3-OH)]^4^ (12:0)
1365.89	1365.64	Penta-acyl	HexN^2^P[12:0 (3-OH)]^2^ [10:0 (3-OH)]^2^ (14:0)
1275.73	1275.49	Tetra-acyl	HexN^2^P^2^[12:0 (3-OH)]^2^ [10:0 (3-OH)] (14:0)
1195.76	1195.54	Tetra-acyl	HexN^2^P[12:0 (3-OH)]^2^ [10:0 (3-OH)] (14:0)

## Supplementary Materials

The following are available online at https://www.mdpi.com/1660-3397/18/12/592/s1, Figure S1: Field emission scanning electron microscopy (FESEM) micrographs of *P. tetraodonis* SY74 (A) and *P. cryohalolentis* SY185 (B) cultures growing at 0.5 °C.; Figure S2. Reflectron MALDI-TOF mass spectrum, recorded in negative polarity, of the *P. arctica* strain SY204b cell pellet. Figure S3. Reflectron MALDI-TOF mass spectrum, recorded in negative polarity, of the cell pellet of *P. cryohalolentis* strain SY185. “P” indicates the phosphate group; Figure S4. Reflectron MALDI-TOF mass spectrum, recorded in negative polarity, of the cell pellet of *P. tetraodonis* strain SY174.

## Abbreviations

core OS	Core oligosaccharide
GC-MS	Gas Chromatography-Mass Spectrometry
Hepta Lip A	Hepta-acylated Lipid A
Hexa Lip A	Hexa-acylated Lip A
LPS	Lipopolysaccharide
MALDI-TOF MS	Matrix Assisted Laser Desorption Ionization-Time of Flight Mass Spectrometry
Penta Lip A	Penta-acylated Lipid A
R-LPS	Rough-type
SWBM	Sea water based medium
S-LPS	Smooth-type LPS
SDS-PAGE	Sodium Dodecyl Sulphate-Polyacrylamide Gel Electrophoresis
Tetra Lip A	Tetra-acylated Lipid A
TLR4/MD-2	Toll-Like Receptor 4/Myeloid Differentiation factor-2
