# Supplementary material for: The Structure of the Lipid A of Gram-Negative Cold-Adapted Bacteria Isolated from Antarctic Environments
Source: Mar Drugs. 2020 Nov 26;18(12):592. doi: 10.3390/md18120592 (PMC7759928; doi:10.3390/md18120592)

Supplementary Material

The Structure of the Lipid A of Gram-Negative Cold-Adapted Bacteria Isolated from Antarctic Environments

Flaviana Di Lorenzo[1]*, Francesca Crisafi[2], Violetta La Cono[2], Michail M Yakimov[2], Antonio Molinaro[1], Alba Silipo[1]*

[1] Department of Chemical Sciences, University of Napoli Federico II, Complesso Universitario Monte S. Angelo, Via Cintia 4, I-80126 Napoli, Italy; flaviana.dilorenzo@unina.it, molinaro@unina.it, silipo@unina.it

[2] Marine Molecular Microbiology & Biotechnology Institute for Biological Resources and Marine Biotechnologies CNR-IRBIM Sede di Messina Spianata San Raineri, 86
98122 Messina, Italy; francesca.crisafi@irbim.cnr.it, violetta.lacono@irbim.cnr.it

* Correspondence: flaviana.dilorenzo@unina.it (F.D.L.); silipo@unina.it (A.S.);

Figure S1

Figure S2

Figure S3

Figure S4

Figure S1. Field emission scanning electron microscopy (FESEM) micrographs of *P. tetraodonis* SY74 (A) and *P. cryohalolentis* SY185 (B) cultures growing at 0.5°C. Images were obtained with microscope Zeiss Merlin (Carl Zeiss, Oberkochen) using the Everhart Thornley SE-detector and the in-lens secondary electron detector in a 50:50 ratio with an acceleration voltage of 5 kV. Contrast and brightness were adjusted with Adobe Photoshop CS5.

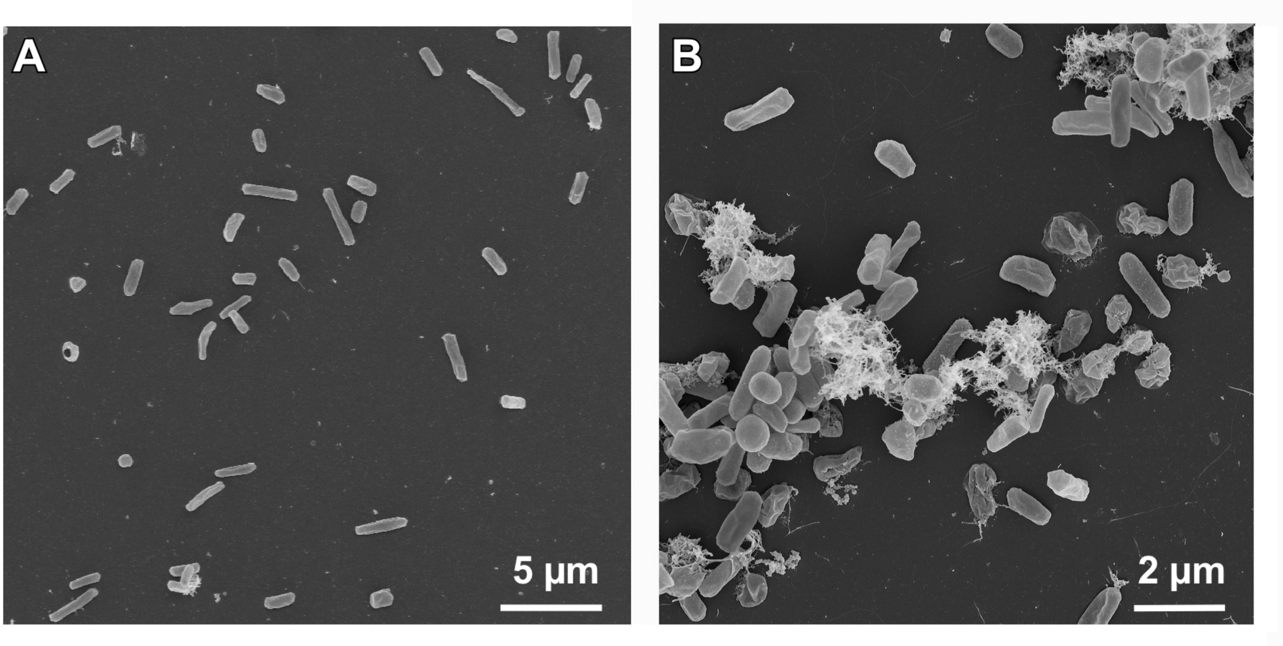

Figure S2. Reflectron MALDI-TOF mass spectrum, recorded in negative polarity, of the *P. arctica* strain SY204b cell pellet. "**P**" indicates the phosphate group.

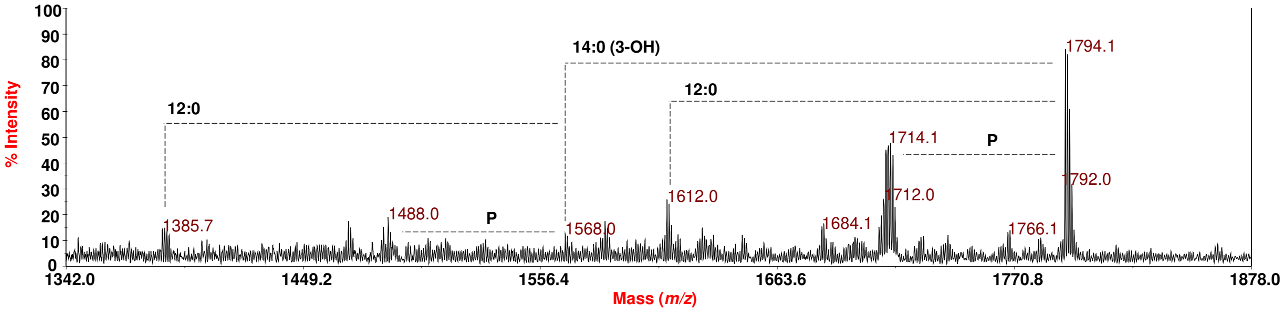

Figure S3. Reflectron MALDI-TOF mass spectrum, recorded in negative polarity, of the cell pellet of *P. cryohalolentis* strain SY185. "**P**" indicates the phosphate group.

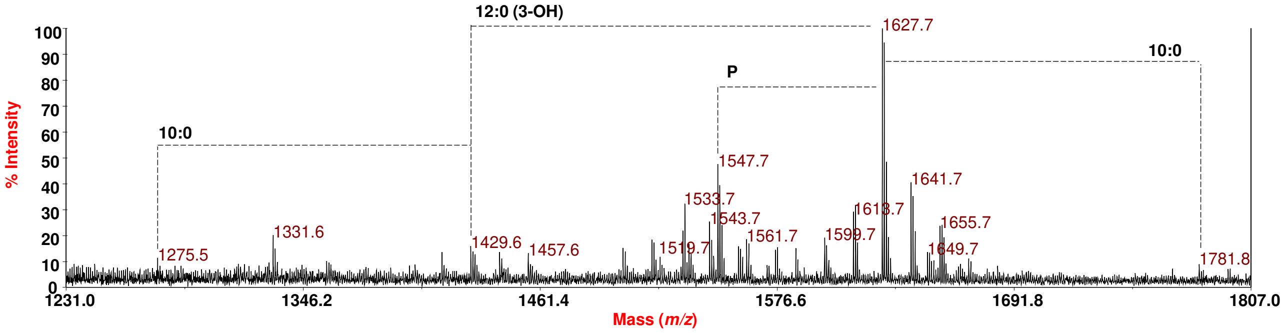

Figure S4. Reflectron MALDI-TOF mass spectrum, recorded in negative polarity, of the cell pellet of *P. tetraodonis* strain SY174. "P" indicates the phosphate group.

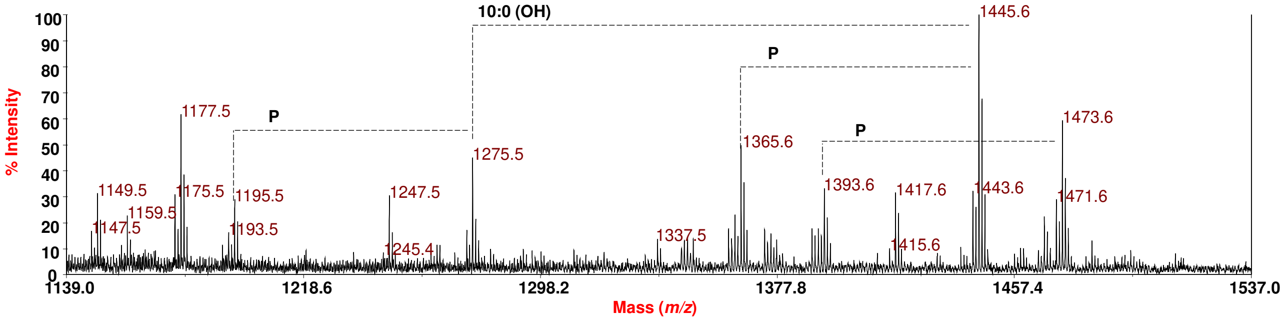

Supplement: Supplementary file 1 [file marinedrugs-18-00592-s001.pdf]
